# Evolution of *African cassava mosaic virus *by recombination between bipartite and monopartite begomoviruses

**DOI:** 10.1186/1743-422X-9-67

**Published:** 2012-03-14

**Authors:** Fidèle Tiendrébéogo, Pierre Lefeuvre, Murielle Hoareau, Mireille A Harimalala, Alexandre De Bruyn, Julie Villemot, Valentin SE Traoré, Gnissa Konaté, Alfred S Traoré, Nicolas Barro, Bernard Reynaud, Oumar Traoré, Jean-Michel Lett

**Affiliations:** 1 Institut de l'Environnement et de Recherches Agricoles (INERA), 01 BP 476, Ouagadougou 01, Burkina Faso; 2 CIRAD, UMR PVBMT, Pôle de Protection des Plantes, Saint-Pierre, Ile de La Réunion 97410, France; 3 CRSBAN/UFR/SVT, Université de Ouagadougou, 03 BP 7021, Ouagadougou 03, Burkina Faso; 4 Faculté des Sciences, Université d'Antananarivo, Antananarivo BP 906, Madagascar; 5 Université de La Réunion, UMR PVBMT, Pôle de Protection des Plantes, Saint-Pierre, Ile de La Réunion 97410, France

**Keywords:** Cassava mosaic disease, *African cassava mosaic virus*, Begomovirus, Evolution, Recombination

## Abstract

**Background:**

Cassava mosaic disease (CMD) is a major constraint on cassava cultivation in Africa. The disease is endemic and is caused by seven distinct cassava mosaic geminiviruses (CMGs), some of them including several variants.

**Findings:**

From cassava leaf samples presenting CMD symptoms collected in Burkina Faso, four DNA-A begomovirus components were cloned and sequenced, showing 99.9% nucleotide identity among them. These isolates are most closely related to *African cassava mosaic virus *(ACMV) but share less than 89% nucleotide identity (taxonomic threshold) with any previously described begomovirus. A DNA-B genomic component, sharing 93% nucleotide identity with DNA-B of ACMV, was also characterized. Since all genomic components have a typical genome organization of Old World bipartite begomoviruses, this new species was provisionally named African cassava mosaic Burkina Faso virus (ACMBFV). Recombination analysis of the new virus demonstrated an interspecies recombinant origin, with major parents related to West African isolates of ACMV, and minor parents related to *Tomato leaf curl Cameroon virus *and *Cotton leaf curl Gezira virus*.

**Conclusion:**

This is the first report of an ACMV-like recombinant begomovirus arisen by interspecific recombination between bipartite and monopartite African begomoviruses.

## Findings

Cassava (*Manihot esculenta *Crantz), the primary food crop in sub-Saharan Africa, is severely affected by cassava mosaic disease (CMD) with losses estimated to up to 19 millions tons (1.9-2.7 billions dollars) annually [1]. CMD is caused by distinct cassava mosaic geminiviruses (CMGs) (family *Geminiviridae*: Genus *Begomovirus*) which are transmitted by the whitefly *Bemisia tabaci *(Genn.) [1]. Whereas begomoviruses are either bipartite with two genomic components (DNA-A and DNA-B) or monopartite with only a DNA-A-like component, all described CMGs are bipartite. Their two circular single stranded DNA molecules (~2.8 kb each) encode a total of eight viral proteins responsible, among other functions, for replication, transcription enhancement, encapsidation and viral movement (see [2] for a review).

CMD is endemic in Africa and seven distinct species are found associated with the disease: *African cassava mosaic virus *(ACMV), *East African cassava mosaic virus *(EACMV), *East African cassava mosaic Cameroon virus *(EACMCV), *East African cassava mosaic Kenya virus *(EACMKV), *East African cassava mosaic Malawi virus *(EACMMV), *East African cassava mosaic Zanzibar virus *(EACMZV) and *South African cassava mosaic virus *(SACMV) [3]. Several strains of the EACMV species are also described throughout Africa [2], many of which resulting from inter- or intra-species recombination [2]. Indeed, it was established that begomoviruses are subject to frequent recombination which provide the viruses with a large sequence space and may promote the adaptation to environmental changes and new ecological niches [4-7]. In contrast to other CMGs, including the EACMV-like viruses (EACMV, EACMCV, EACMKV, EACMMV, EACMZV), SACMV, *Sri Lanka cassava mosaic virus *(SLCMV) and *Indian cassava mosaic virus *(ICMV), the genomes of ACMV isolates present little genetic variation and do not display evidence of recombination, suggesting that ACMV has evolved as a distinct lineage (reviewed by [2]).

In West Africa, cassava is frequently cultivated in association with several other crops such as vegetables (tomato, pepper, okra), tubers (cassava, sweet potato, yam), and cereals. In August/September 2008, leaves from cassava plants presenting CMD symptoms were collected from such a field around Ouagadougou (12°23'31.42"N; 1°30'19.40"W; Burkina Faso). Total DNA was extracted using DNeasy^® ^Plant Minikit (Qiagen) and tested for the presence of begomoviruses using polymerase chain reaction (PCR) with degenerate primers representative of either DNA-A [8] or DNA-B [9].

Ten samples showing mosaic symptoms were found to be positive for the presence of begomovirus DNA using PCR. The primary results showed the presence of ACMV and EACMV-UG in seven and one samples respectively [10]. From two of those samples (BF127 and BF128), full-length viral genomes were successfully amplified by rolling-circle amplification (RCA) [11]. The amplified DNAs were digested with endonucleases *Bam*HI, *Pst*I, *Apa*I or *Nco*I and the DNA fragments of the expected size (~2.8 kb) were cloned into pGEM^® ^vectors (Promega Biotech). Cloned genome components were sequenced by Macrogen Inc. (South Korea) by primer walking and genomic components were assembled using the DNAMAN software (version 5.2.9; Lynnon, Quebec, Canada). Four full-length DNA-A nucleotide sequences, one from sample BF127 (BF:Oua:BF127:08) and three from sample BF128 (BF:Oua:BF128A:08; BF:Oua:BF128B:08; BF:Oua:BF128C:08), as well as one full-length DNA-B nucleotide sequence from sample BF127 (BF:BF127:08), were obtained (Table 1). The sequences were deposited in the EMBL database under the accession numbers HE616777 to HE616781.

**Table 1:** Name, acronym and accession numbers of begomovirus isolates reported in this study

Name	Acronym	DNA molecule, size (nt) and accession numbers	
		
		DNA-A	DNA-B
African cassava mosaic Burkina Faso virus- [Burkina Faso:Ouagadougou:BF127:2008]	ACMBFV-[BF:Oua:BF127:08]	2770 (HE616777)	2737 (HE616778)
African cassava mosaic Burkina Faso virus- [Burkina Faso:Ouagadougou:BF128A:2008]	ACMBFV-[BF:Oua:BF128A:08]	2768 (HE616779)	
African cassava mosaic Burkina Faso virus- [Burkina Faso:Ouagadougou:BF128B:2008]	ACMBFV-[BF:Oua:BF128B:08]	2768 (HE616780)	
African cassava mosaic Burkina Faso virus- [Burkina Faso:Ouagadougou:BF128C:2008]	ACMBFV-[BF:Oua:BF128C:08]	2768 (HE616781)	

The cloned genomes showed an organization typical of bipartite begomoviruses from the Old World, *i.e*. DNA-A components (2768 to 2770 nt each) with two ORFs on the viral strand (AV2/MP and AV1/CP), and four on the complementary-sense strand (AC1/Rep, AC2/TrAP, AC3/REn and AC4) and a DNA-B component (2737 nt) with one ORF on the viral-sense (BV1/MP), and one on the complementary-sense (BC1/NSP). Sequences were aligned with representative isolates of begomoviruses using the Muscle [12] and ClustalW [13] alignment tools implemented in the MEGA 5 software [14]. Pairwise sequence comparisons performed with MEGA 5 (with pairwise deletion of gaps) showed that the four genomic DNA-A components are genetically related with 99.9% identity between one another. The four DNA-A sequences displayed highest nucleotide identity (87%) with ACMV-[Côte d'Ivoire:1999] (Accession n° AF259894) and ACMV-[Nigeria:1990] (X17095). The DNA-B sequence displayed 93% nucleotide identity with ACMV-[Nigeria:Ogo:2002] (AJ427911). Based on the species demarcation criterion of the genus *Begomovirus *(< 89% nucleotide identity for DNA-A) [15], the begomovirus molecularly characterised in this work could be considered as a novel species, provisionally named African cassava mosaic Burkina Faso virus (ACMBFV).

The putative Rep protein binding site of the four ACMBFV isolates included a single core binding site (**GGGGT**) with a potential inverted repeat (**GGACC**), and the corresponding iteron-related domain (IRD) was identified to be MAPPRK**FRIN**. These features are different of those from previously described ACMV isolates (Iteron: **GGAGA **and IRD: MRTPR**FRIQ **or MRTPR**FRVQ**), but are similar to those found in isolates of *Tomato leaf curl Cameroon virus *(ToLCCMV) and *Cotton leaf curl Gezira virus *(CLCuGV), two monopartite begomoviruses [16,17]. Interestingly, the presence of the same iteron (GGGGT) in the intergenic region of the ACMBFV DNA-B is consistent with its trans-replication by the Rep protein encoded by the ACMBFV DNA-A component.

As these results suggested that the ACMBFV isolates could have a recombinant origin, a recombination analysis was performed on a set of 234 sequences, with 222 sequences representing the whole Old World begomovirus diversity, ten *Curtovirus *and two *Topocuvirus *sequences. The data set was retrieved from public sequence databases using taxonomy browser (http://www.ncbi.nlm.nih.gov) on September 2011 and aligned as described above with our sequences. Detection of potential recombinant sequences, identification of likely parental sequences, and localization of possible recombination breakpoints was carried out using RDP [18], GENECONV [19], BOOTSCAN [20], MAXIMUM CHI SQUARE [21], CHIMAERA [20], SISTER SCAN [22] and 3Seq [23] recombination detection methods as implemented in RDP3 [24]. The analysis was performed so that only triplets of sequences involving at least two CMG sequences were considered. Default settings for the different detection methods and a Bonferroni corrected *P*-value cut-off of 0.05 where used. Only events detected with 3 methods or more were accepted.

**Figure 1:**
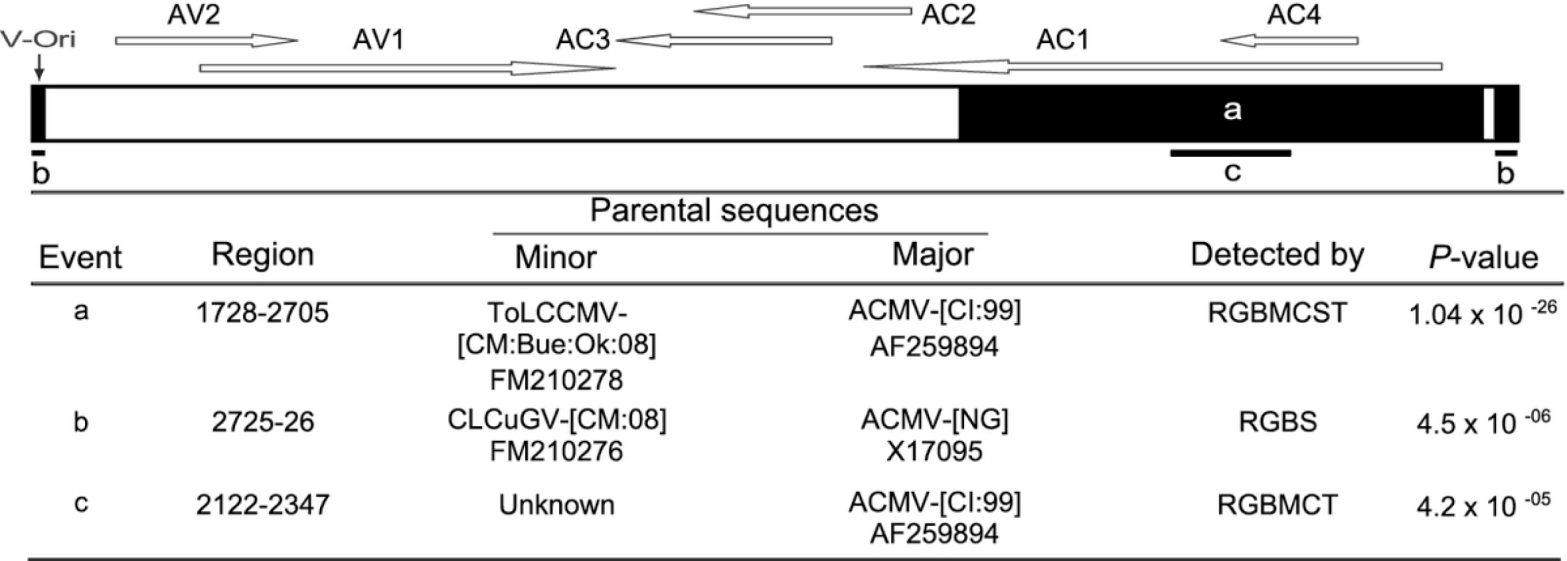
**Recombinant regions (a, b and c) detected within the isolates of *African cassava mosaic Burkina Faso virus *using RDP3**. The genome at the top of the figure corresponds to the schematic representation of sequence below. Region coordinates are nucleotide positions of detected recombination breakpoints. Wherever, possible parental sequences are identified. "Major" and "Minor" parents are sequences that were used, along with the indicated recombinant sequence to identify recombination. Whereas for each identified event the minor parent is apparently the contributor of the sequence within the indicated region, the major parent is the apparent contributor of the rest of the sequence. Note that the identified "parental sequences" are not the actual parents but are simply those sequences most similar to the actual parents in the analysed dataset. Recombinant regions and parental viruses were identified using the RDP (R), GENECONV (G), BOOTSCAN (B), MAXIMUM CHI SQUARE (M), CHIMAERA (C), SISTER SCAN (S) and 3Seq (T) methods.

Three distinct recombination events (a, b and c; Figure 1) were detected within the genomic DNA-A of ACMBFV, whereas no recombination was detected within the DNA-B. Recombination event (a) involves a major parent related to the Ivory Coast isolate of the bipartite ACMV (ACMV-[Côte d'Ivoire:1999]), and a minor parent related to the Cameroon isolate of the monopartite ToLCCMV (ToLCCMV-[Cameroon:Buea:Okra:2008]). Event (b) detected in the intergenic region occurred between ACMV-[Nigeria] as a major parent and CLCuGV-[Cameroon:2008], another monopartite begomovirus described in Cameroon on okra as a minor parent. The event (c) was detected into the event (a) between the same major parent and an unknown minor parent (undescribed or already extinct virus).

**Figure 2:**
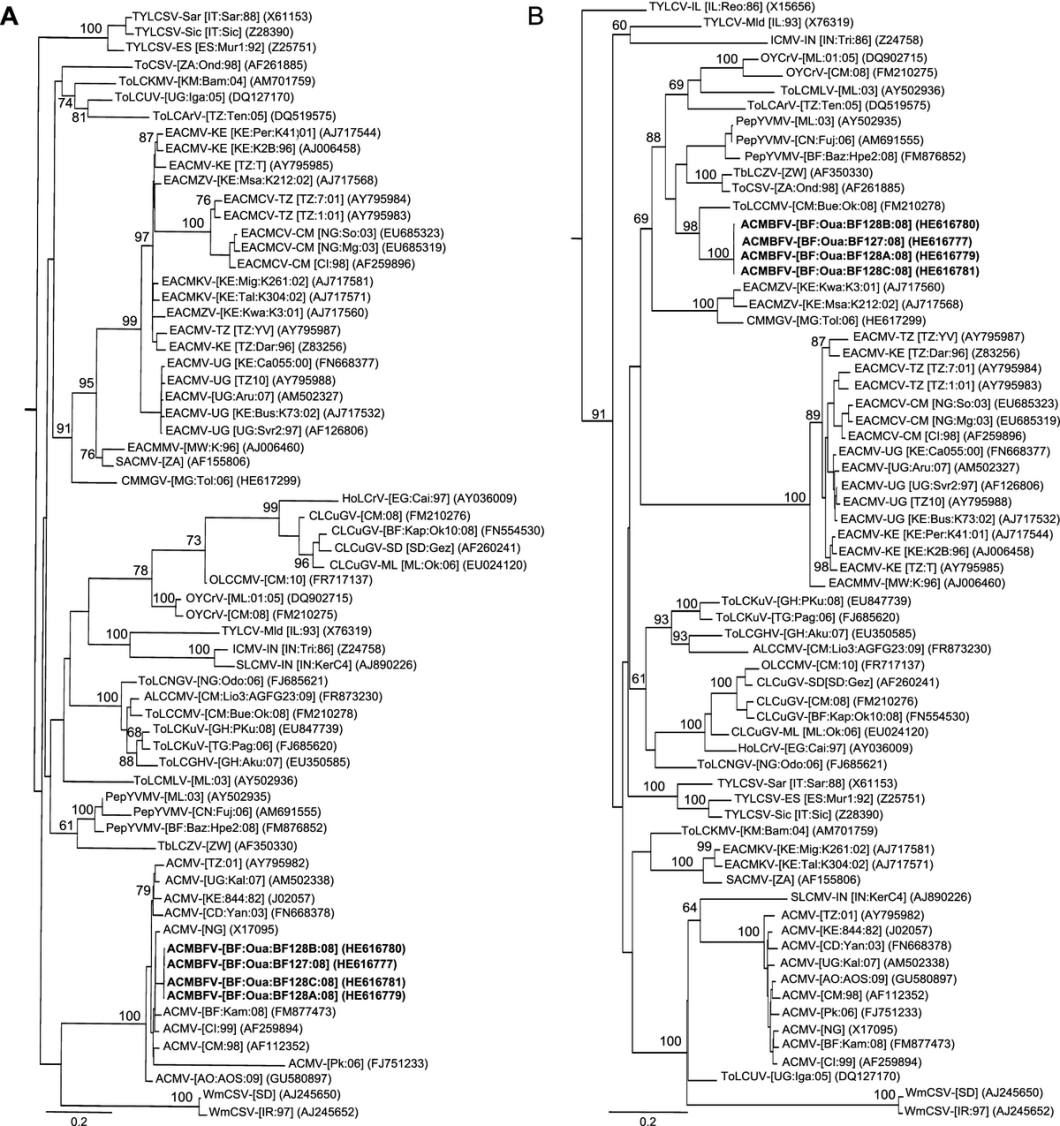
**Maximum likelihood trees based on the alignment of the sequences of four *African cassava mosaic Burkina Faso virus *isolates (in bold; see Table 1 for isolates names and acronyms), plus additional DNA-A and DNA-A-like sequences originated from Africa and the Mediterranean region**. In (**A**) the non-recombinant portion (ORFs: AV2, AV1, AC2 and AC3; see Figure 1) of the genome and in (**B**) the recombinant portion (covering 90% of 3' portion of Rep, entire AC4 ORF and IR; see Figure 1). Begomovirus acronyms used are described in the Additional file 1: Table S1 according to Fauquet et al. [15] isolate descriptors.

**Figure 3:**
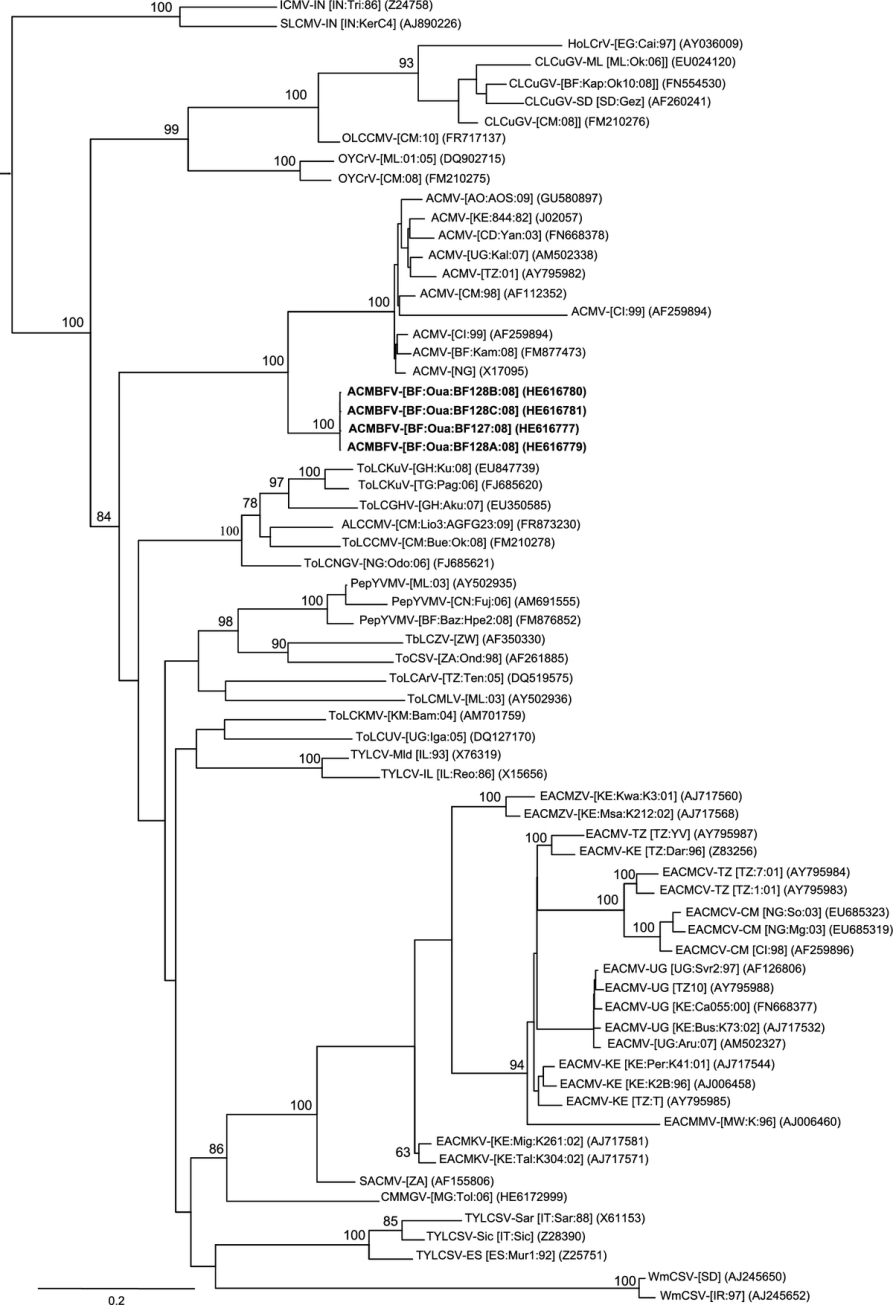
**Maximum likelihood tree based on the complete DNA-A sequences of four *African cassava mosaic Burkina Faso virus *isolates (in bold; see Table 1 for isolates name and acronyms), plus additional DNA-A and DNA-A-like sequences originated from Africa and the Mediterranean region**. Begomovirus acronyms used are described in the Additional file 1: Table S1.

**Figure 4:**
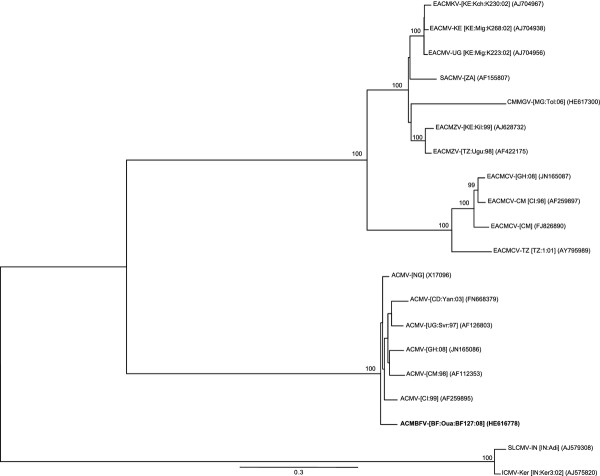
**Maximum likelihood tree based on the complete DNA-B sequence of *African cassava mosaic Burkina Faso virus *(in bold; see Table 1 for name and acronym), plus DNA-B additional sequences from Africa and India**. Begomovirus acronyms used are described in the Additional file 1: Table S1.

Maximum likelihood (ML) phylogenetic trees (with 100 bootstrap replicates) were constructed from full DNA components as well as alignments of both parts of the recombination event (a) using PHYML v3.0. [25] and the model of sequence evolution selection procedure implemented in RDP3 [24]. Trees were visualized using FigTree v1.3.1 software (available at http://tree.bio.ed.ac.uk/software/figtree/). Consistently, phylogenetic analyses performed with the non-recombinant portion of the genome (nt 27-1727 relative to ACMBFV-[BF:Oua:BF127:08]; covering AV2, AV1, AC2 and AC3) placed ACMBFV in the cluster of ACMV (Figure 2A). With the recombinant portion (nt 1728-26 relative to ACMBFV-[BF:Oua:BF127:08]; covering 90% of 3' portion of Rep, entire AC4 and IR) the same analysis placed ACMBFV in a cluster with ToLCCMV-[CM:Bue:Ok:08] (Figure 2B). Taken together, these results confirm the recombinant nature of the ACMBFV, with the majority of the genome (61.5%) derived from a major ACMV parent, while the rest of the genome (38.5%) is related to ToLCCMV-[CM:Bue:Ok:08] and CLCuGV-[CM:08] minor parents. As expected, phylogenetic reconstruction performed with the DNA-A sequences, using GTR + I + G4 as the best fit model of evolution, placed ACMBFV outside the cluster of ACMV isolates, due to its recombinant nature (Figure 3). A similar analysis performed with the DNA-B sequence of ACMBFV under GTR + G4 evolutionary model placed it within the cluster of ACMV isolates (Figure 4). In order to assess its biological features, the infectivity of our recombinant DNA-A and the need of a cognate DNA-B of ACMV type should be investigated in the future.

In conclusion, we describe for the first time an ACMV-like recombinant begomovirus arisen by interspecific recombination between bipartite and monopartite African begomoviruses. Although current data suggest that ACMV has evolved as a distinct lineage, our results prove that the ACMV genotype is also prone to recombination. Our study confirms that West Africa is likely a centre of begomovirus diversity [17], where recombination seems to play a major role in the evolution of food and vegetable crops infecting begomoviruses.

## Competing interests

The authors declare that they have no competing interests.

## Authors' contributions

FT, VSET and OT collected samples. FT, MH, MAH, ADB and JV cloned and sequenced the viruses; FT, PL and JML analysed the data and prepared the manuscript. JML, OT, NB, GK, AST, VSET and BR secured funding for the project's execution, and provided ideas and comments during manuscript preparation. All authors read and approved the final version of the manuscript.

## Supplementary Material

Additional file 1 **Table S1**. Description of begomovirus acronyms used in the phylogenetic trees. Click here for file
