# Supplementary material for: Evolution of African cassava mosaic virus by recombination between bipartite and monopartite begomoviruses
Source: Virol J. 2012 Mar 14;9:67. doi: 10.1186/1743-422X-9-67 (PMC3328289; doi:10.1186/1743-422X-9-67)
Supplement: Additional file 1 — Table S1. Description of begomovirus acronyms used in the phylogenetic trees. [file 1743-422X-9-67-S1.DOC]

**Supplementary Table 1**: Description of begomovirus acronyms used in the phylogenetic trees

| **Name** | **Acronym** | **Accession numbers** | |
| --- | --- | --- | --- |
|  |  | **DNA-A** | **DNA-B** |
| African cassava mosaic virus-[Angola:AOS:2009] | ACMV-[AO:AOS:09] | GU580897 |  |
| African cassava mosaic virus-[Burkina Faso:Kamboinsé:2008] | ACMV-[BF:Kam:08] | FM877473 |  |
| African cassava mosaic virus-[Cameroon:1998] | ACMV-[CM:98] | AF112352 |  |
| African cassava mosaic virus-[Cameroon:1998] | ACMV-[CM:98] |  | AF112353 |
| African cassava mosaic virus-  [Côte-d’Ivoire:1999] | ACMV-[CI:99] | AF259894 |  |
| African cassava mosaic virus-  [Côte-d’Ivoire:1999] | ACMV-[CI:99] |  | AF259895 |
| African cassava mosaic virus-[Democratic Republic of the Congo:Yangambi:2003] | ACMV-[CD:Yan:03] | FN668378 |  |
| African cassava mosaic virus-[Democratic Republic of the Congo:Yangambi:2003] | ACMV-[CD:Yan:03] |  | FN668379 |
| African cassava mosaic virus-[Ghana:2008] | ACMV-[GH:08] |  | JN165086 |
| African cassava mosaic virus-[Kenya:844:1982] | ACMV-[KE:844:82] | J02057 |  |
| African cassava mosaic virus-[Nigeria] | ACMV-[NG] | X17095 |  |
| African cassava mosaic virus-[Nigeria] | ACMV-[NG] |  | X17096 |
| African cassava mosaic virus-[Pakistan:2006] | ACMV-[Pk:06] | FJ751233 |  |

**Supplementary Table 1: continued**

| African cassava mosaic virus-[Tanzania:2001] | ACMV-[TZ:01] | AY795982 | |  | |
| --- | --- | --- | --- | --- | --- |
| African cassava mosaic virus-[Uganda:Kalangala:2007] | ACMV-[UG:Kal:07] | AM502338 | |  | |
| African cassava mosaic virus-[Uganda:Severe:1997] | ACMV-[UG:Svr:97] |  | | AF126803 | |
| Agaratum leaf curl Cameroon virus-[Cameroon:Lio3:AGFG23:2009] | ALCCMV-[CM:Lio3:AGFG23:09] | FR873230 | |  | |
| Cassava mosaic Madagascar virus-[Madagascar:Toliary:2006] | CMMGV-[MG:Tol:06] | HE617299 |  | | |
| Cassava mosaic Madagascar virus-[Madagascar:Toliary:2006] | CMMGV-[MG:Tol:06] |  | | | HE617300 |
| Cotton leaf curl Gezira virus-  [Burkina Faso:Kampala:Okra10:2008] | CLCuGV-[BF:Kap:Ok10:08] | FN554530 | | |  |
| Cotton leaf curl Gezira virus-[Cameroon:2008] | CLCuGV-[CM :08] | FM210276 | | |  |
| Cotton leaf curl Gezira virus-  Mali [Mali:Okra:2006] | CLCuGV-  ML [ML:Ok:06] | EU024120 | | |  |
| Cotton leaf curl Gezira virus-  Sudan [Sudan:Gezira] | CLCuGV-SD [SD:Gez] | AF260241 | | |  |
| East African cassava mosaic Cameroon virus [Cameroon] | EACMCV-[CM] |  | | | FJ826890 |
| East African cassava mosaic Cameroon virus-[Ghana:2008] | EACMCV-[GH:08] |  | | | JN165087 |
| East African cassava mosaic Cameroon virus-Cameroon  [Côte d’Ivoire:1998] | EACMCV-CM [CI:98] | AF259896 | | |  |

**Supplementary Table 1: continued**

| East African cassava mosaic Cameroon virus-Cameroon  [Côte d’Ivoire:1998] | EACMCV-  CM [CI:98] |  | AF259897 |
| --- | --- | --- | --- |
| East African cassava mosaic Cameroon virus-Cameroon [Nigeria:Mg:2003] | EACMCV-CM [NG:Mg:03] | EU685319 |  |
| East African cassava mosaic Cameroon virus-Cameroon[Nigeria:So:2003] | EACMCV-CM[NG:So:03] | EU685323 |  |
| East African cassava mosaic Cameroon virus-Tanzania [Tanzania:1:2001] | EACMCV-TZ [TZ:1:01] | AY795983 |  |
| East African cassava mosaic Cameroon virus-Tanzania [Tanzania:1:2001] | EACMCV-TZ [TZ:1:01] |  | AY795989 |
| East African cassava mosaic Cameroon virus-Tanzania [Tanzania:7:2001] | EACMCV-TZ [TZ:7:01] | AY795984 |  |
| East African cassava mosaic Kenya virus-[Kenya:Kehancha:K230:2002] | EACMKV-[KE:Kch:K230:02] |  | AJ704967 |
| East African cassava mosaic Kenya virus-[Kenya:Migori:K261:2002] | EACMKV-[KE:Mig:K261:02] | AJ717581 |  |
| East African cassava mosaic Kenya virus-[Kenya:Tala:K304:2002] | EACMKV-[KE:Tal:K304:02] | AJ717571 |  |
| East African cassava mosaic Malawi virus-[Malawi:K:1996] | EACMMV-[MW:K:96] | AJ006460 |  |
| East African cassava mosaic virus-[Uganda:Arua:2007] | EACMV-[UG:Aru:07] | AM502327 |  |
| East African cassava mosaic virus-Kenya [Kenya:K2B:1996] | EACMV-  KE [KE:K2B:96] | AJ006458 |  |

**Supplementary Table 1: continued**

| East African cassava mosaic virus-Kenya [Kenya:Migori:K268:2002] | EACMV-  KE [KE:Mig:K268:02] |  | AJ704938 |
| --- | --- | --- | --- |
| East African cassava mosaic virus-Kenya [Kenya:Perani:K41:2001] | EACMV-KE [KE:Per:K41:01] | AJ717544 |  |
| East African cassava mosaic virus-Kenya [Tanzania:Dar Es Salaam:1996] | EACMV-  KE [TZ:Dar:96] | Z83256 |  |
| East African cassava mosaic virus-Kenya [Tanzania:T] | EACMV-KE [TZ:T] | AY795985 |  |
| East African cassava mosaic virus-Tanzania [Tanzania:YV] | EACMV-TZ [TZ:YV] | AY795987 |  |
| East African cassava mosaic virus-Uganda [Kenya:Busia:K73:2002] | EACMV-UG [KE:Bus:K73:02] | AJ717532 |  |
| East African cassava mosaic virus-Uganda [Kenya:Ca055:2000] | EACMV-UG [KE:Ca055:00] | FN668377 |  |
| East African cassava mosaic virus-Uganda [Kenya:Migori:K223:2002] | EACMV-UG [KE:Mig:K223:02] |  | AJ704956 |
| East African cassava mosaic virus-Uganda [Tanzania:10] | EACMV-UG [TZ10] | AY795988 |  |
| East African cassava mosaic virus-Uganda [Uganda:Severe2:1997] | EACMV-UG [UG:Svr2:97] | AF126806 |  |
| East African cassava mosaic Zanzibar virus-[Kenya:Kilifi:1999] | EACMZV-[KE:Kil:99] |  | AJ628732 |
| East African cassava mosaic Zanzibar virus-[Kenya:Kwakadzengo:K3:2001] | EACMZV-[KE:Kwa:K3:01] | AJ717560 |  |
| East African cassava mosaic Zanzibar virus-[Kenya:Msambweni:K212:2002] | EACMZV-[KE:Msa:K212:02] | AJ717568 |  |
| East African cassava mosaic Zanzibar virus-[Tanzania:Uguja:1998] | EACMZV-[TZ:Ugu:98] |  | AF422175 |

Supplementary Table 1 : Continued

| Hollyhock leaf crumple virus-[Egypt:Cairo:1997] | HoLCrV-[EG:Cai:97] | AY036009 |  |
| --- | --- | --- | --- |
| Indian cassava mosaic virus-India [India:Trivandrum:1986] | ICMV-IN [IN:Tri:86] | Z24758 |  |
| Indian cassava mosaic virus-Kerala [India:Kerala 3:2002] | ICMV-Ker [IN:Ker3:02] |  | AJ575820 |
| Okra leaf curl Cameroon virus-[Cameroon:10] | OLCCMV-[CM:10] | FR717137 |  |
| Okra yellow crinkle virus-[Camroon:2008] | OYCrV-[CM:08] | FM210275 |  |
| Okra yellow crinkle virus-[Mali:01:2005] | OYCrV-[ML:01:05] | DQ902715 |  |
| Pepper yellow vein Mali virus-[Burkina Faso:Bazega:hot pepper2:  2008] | PepYVMV-[BF:Baz:Hpe2:08] | FM876852 |  |
| Pepper yellow vein Mali virus-[China:Fujian:2006] | PepYVMV-[CN:Fuj:06] | AM691555 |  |
| Pepper yellow vein Mali virus-[Mali:2003] | PepYVMV-[ML:03] | AY502935 |  |
| South African cassava mosaic virus-[South Africa] | SACMV-[ZA] | AF155806 |  |
| South African cassava mosaic virus-[South Africa] | SACMV-[ZA] |  | AF155807 |
| Sri Lankan cassava mosaic virus-India [India:Adivaram] | SLCMV-IN [IN:Adi] |  | AJ579308 |
| Sri Lankan cassava mosaic virus-India [India:Kerala C4] | SLCMV-IN [IN:KerC4] | AJ890226 |  |
| Tobacco leaf curl Zimbabwe virus-[Zimbabwe] | TbLCZV-[ZW] | AF350330 |  |
| Tomato leaf curl Arusha virus-[Tanzania:Tengelu:2005]: | ToLCArV-[TZ:Ten:05] | DQ519575 |  |

Supplementary Table 1 : Continued

| Tomato leaf curl Cameroon virus-[Cameroon:Buea:Okra:2008] | ToLCCMV-[CM:Bue:Ok:08] | FM210278 |  |
| --- | --- | --- | --- |
| Tomato leaf curl Comoros virus-[Comoros:Bambas:2004] | ToLCKMV-[KM:Bam:04] | AM701759 |  |
| Tomato leaf curl Ghana virus-[Ghana:Akumadan:2007] | ToLCGHV-[GH:Aku:07] | EU350585 |  |
| Tomato leaf curl Kumasi virus-[Ghana:Kumasi:2008] | ToLCKuV-[GH:Ku:08] | EU847739 |  |
| Tomato leaf curl Kumasi virus-[Togo:Pagouda:2006] | ToLCKuV-[TG:Pag:06] | FJ685620 |  |
| Tomato leaf curl Mali virus-[Mali:2003] | ToLCMLV-[ML:03] | AY502936 |  |
| Tomato leaf curl Nigeria virus-[Nigeria:Odogbo:2006] | ToLCNGV-[NG:Odo:06] | FJ685621 |  |
| Tomato leaf curl stunt virus-[South Africa:Onderberg:1998] | ToCSV-[ZA:Ond:98] | AF261885 |  |
| Tomato leaf curl Uganda virus-[Uganda:Iganga:2005] | ToLCUV-[UG:Iga:05] | DQ127170 |  |
| Tomato leaf curl virus-Tomato [Australia] | ToLCV-To [AU] | S53251 |  |
| Tomato yellow leaf curl Sardinia virus-Sardinia [Italy:Sardinia:1988] | TYLCSV-Sar [IT:Sar:88] | X61153 |  |
| Tomato yellow leaf curl Sardinia virus-Sicily [Italy:Sicily] | TYLCSV-Sic [IT:Sic] | Z28390 |  |

Supplementary Table 1 : Continued

| Tomato yellow leaf curl Sardinia virus-Spain [Spain:Murcia 1:1992] | TYLCSV-ES [ES:Mur1:92] | Z25751 |  |
| --- | --- | --- | --- |
| Tomato yellow leaf curl virus-Israel[Israel:Rehovot:1986] | TYLCV-IL[IL:Reo:86] | X15656 |  |
| Tomato yellow leaf curl virus-Mild [Israel:1993] | TYLCV-Mld [IL:93] | X76319 |  |
| Watermelon chlorotic stunt virus-[Iran:1997] | WmCSV-[IR:97] | AJ245652 |  |
| Watermelon chlorotic stunt virus-[Sudan] | WmCSV-[SD] | AJ245650 |  |
